# Review and Simulation of Counter-UAS Sensors for Unmanned Traffic Management

**DOI:** 10.3390/s22010189

**Published:** 2021-12-28

**Authors:** Juan A. Besada, Ivan Campaña, David Carramiñana, Luca Bergesio, Gonzalo de Miguel

**Affiliations:** Information Processing and Telecommunications Center, Universidad Politécnica de Madrid, 28040 Madrid, Spain; ivan.campana@upm.es (I.C.); d.carraminana@upm.es (D.C.); luca.bergesio@upm.es (L.B.); gonzalo.demiguel@upm.es (G.d.M.)

**Keywords:** counter-UAS sensors, unmanned traffic management, review, simulation models

## Abstract

Noncollaborative surveillance of airborne UAS (Unmanned Aerial System) is a key enabler to the safe integration of UAS within a UTM (Unmanned Traffic Management) ecosystem. Thus, a wide variety of new sensors (known as Counter-UAS sensors) are being developed to provide real-time UAS tracking, ranging from radar, RF analysis and image-based detection to even sound-based sensors. This paper aims to discuss the current state-of-the art technology in this wide variety of sensors (both academically and commercially) and to propose a set of simulation models for them. Thus, the review is focused on identifying the key parameters and processes that allow modeling their performance and operation, which reflect the variety of measurement processes. The resulting simulation models are designed to help evaluate how sensors’ performances affect UTM systems, and specifically the implications in their tracking and tactical services (i.e., tactical conflicts with uncontrolled drones). The simulation models cover probabilistic detection (i.e., false alarms and probability of detection) and measurement errors, considering equipment installation (i.e., monostatic vs. multistatic configurations, passive sensing, etc.). The models were integrated in a UTM simulation platform and simulation results are included in the paper for active radars, passive radars, and acoustic sensors.

## 1. Introduction

The use of UAVs (Unmanned Aerial Vehicles), or as they are commonly known, drones, has increased in recent years. Initially, these aircraft were used as military technology, especially for security and monitoring purposes, but today, many companies and private users are using UAVs in their daily lives. These nonmilitary drones are used by citizens for recreational activities, such as video recording or taking high-resolution photos, and by companies for observation, transportation, field monitoring, traffic monitoring, fire protection and border patrol, among many other uses [1]. In addition to their widespread use for actions such as those described above, UAVs can be hacked and used to commit crimes, such as espionage, smuggling or even attacks.

For all these reasons, drone detection is necessary to check their presence near critical areas or infrastructures, and if a drone’s behavior is appropriate and compatible with other air operations (of manned aircraft or other drones). There are many different technologies enabling drone detection, localization, and tracking, including cooperative and noncooperative sensors. This paper focuses on this second type of sensors.

Over the past five years, significant research efforts have been made to detect and counter UAVs, and the main physical operating principles of the different technologies being used are clearly described in [2]. Noncooperative sensors include active and passive radar detection techniques, detection through UAVs radio frequency signals, detection by acoustics signals, image detection and detection by merging these techniques or data fusion. 

In this contribution, we go a little further in the analysis of these technologies. In addition to describing some of the most interesting literature proposals and commercial products in the state of the art, we define a collection of simulation models, usable for some of those technologies and expandable to others, to be potentially usable for:

(a) Comparative assessment of potential systems deployment in a given position.
(b) Analysis of integrated sensing solutions/data fusion approaches for C-UAS.
(c) Analysis through simulation of the potential integration of the measurements from those sensors in UTM tactical chains, specifically to test the associated implications in their tracking and tactical services.

In any case, the paper focuses on modeling the sensing processes for the different technologies, which would be a prerequisite for any of the previously described analyses. Finally, there are plenty of models of radar, RF, vision, and acoustic sensors. Here, we try to select, parameterize, and summarize those of real application for the detection of small drones in civilian applications (for UTM).

The paper is structured as follows: In the second section of this paper, we describe in detail some of these sensing technologies, covering both the academic literature in the area and the fast-evolving commercial scenario. Meanwhile, the third section is devoted to deriving the simulation models of some of these sensors. This simulation models are to be incorporated in the UTM simulator described in [3]. The fourth section summarizes simulation results for some of the previous sensors, enabling a comparison of their main sensing features and performances, and finally, Section 5 concludes the paper, providing some insights on future work.

## 2. Review of the State-of-the-Art Technology

In this section, we summarize the different detection technologies. Section 2.1, Section 2.2, Section 2.3, Section 2.4, Section 2.5 and Section 2.6 describe solutions in the literature and some of the commercial solutions (if available). In the case of Section 2.6, it is important to note that it focuses on the use of fusion approaches making use of different sensing technologies. Therefore, quite often, a commercial solution will be described in several of the following sections, once per sensor type, and again when talking about integrated sensing and fusion C-UAS systems. Finally, Section 2.7 includes a comparative summary of technologies requirements, expected performance and limitations.

### 2.1. Active Detection Radars

Radars have several advantages in detecting aircraft compared with other sensors in terms of weather independency, day and night operation capability, technology development, and capacity to measure range and velocity simultaneously. A big challenge with UAVs is that they have very small radar cross sections (RCS), and they fly at lower altitudes and lower speeds compared to larger aircrafts [4]. Regular radar systems typically aim to detect air targets of medium and large size (RCS larger than 1 m^2^). In addition, due to its low speed, Doppler processing (Moving Target Indication/Detection) is not so effective. In the literature [5], there are several types of radar used for detection, tracking and classification of drones, such as mmWave Radar or Ultrawide-Band Radar, which can be classified in two main categories: active detection and passive detection radars. In this section, we focus on active detection radars, while the next section describes passive radars.

Conventionally, there are two possible ways to increase the distance and azimuth resolution of active radar detection systems in the case of UAVs operations: using higher frequency carriers or utilizing multiple input multiple output (MIMO) beamforming antennas.

To use shorter a wavelength, K-band, X-band and W-band frequency modulated continuous wave (FMCW) radars are specifically designed for UAV detection. The selection of carrier frequency for UAV detection radar should be higher than 6 GHz (K-band), as in [6], where it is verified the ability of radars to detect small, slow, and low-flying targets. There are two important factors to be considered for the use of radars to detect airborne threats: the target to be detected and the radar itself. When a radar is used to detect small and slow targets, the limiting factor is the RCS, so in that work, “mini-UAVs” were treated as a Medium of Airborne Attack (MoAA), and it was concluded that radars working in the K-band are the ones that best detect “mini-UAVs” due to their dimensions and radar sections. These radars offer optimal accuracy for measuring the coordinates of the targets being detected and small antenna dimensions.

Other approaches use multiple antennas following a MIMO approach. The advantage of this approach is in its applicability to a radar system with lower carrier frequencies, as in [7,8]. A Holographic RadarTM (HR) with a 2D antenna array and an appropriate signal processing is used in [7]. This signal processing can create a multibeam, 3D, wide-area, staring surveillance sensor, which is able to achieve high detection sensitivity, and provide fine Doppler resolution, with update rates of fractions of a second. The ability to remain continuously on targets throughout the entire search volume enables the detection of small targets, such as UAVs, against a moving background. The system uses a 32-by-8 element L-Band receiver array. As the radar has a high detection sensitivity, it can detect small drones and other small moving targets as birds. Thus, it is necessary to have a stage of processing to discriminate the UAV from other objects. In this case, a machine learning decision tree classifier is used to reject small objects while maintaining a high probability of detection for the drone. A similar study is presented in [8], where a ubiquitous frequency modulated continuous wave (FMCW) radar system working at 8.75 GHz (X-band) with PC-based signal processor can detect a micro-UAV at a range of 2 km with an excellent range–speed resolution.

The advances in computation enable another type of radar described in the literature for this application, the software-defined radar (SDR) [9]. This radar is a multiband, multimode, software-defined radar that consists of a hardware-based platform and software-based platform. It is multiband because the module allows the selection of S-, X- and K-bands, while it is multimode because of the capacity of selecting waveforms of CW, Pulse, FMCW and LFM Chirp. The detection results of this system show that the detection of the SDR platform if successfully performed in real-time operations, so it can be used for air safety applications by detecting and warning of the threat UAVs.

An example of mmWave Radar with a precise detection and 3D localization system for drones can be observed in [10]. The positions of drones are estimated from spatial heatmaps of the received radar signals, obtained by applying a super-resolution algorithm. These positions are improved analyzing the micro-Doppler effect, which is generated by the rotating propellers. This radar presents a novel Gaussian Process Regression model to compensate for systematic biases in the radar data. 

Finally, another way of detecting UAVs using radars is by means of the Multistatic Forward Scattering Radar (FSR) [11]. The most important principle of a FSR for target detection is the use of the shadow field. When the diffraction angle, which is the angle between the direction of the transmitter–target and the direction of the target–receiver, is approximately zero, the shadow field can be observed at the receiving point. This shadow field is considered in narrow regions where the diffraction angle is approximately zero, causing the forward scatter radar section to increase considerably compared to monostatic radar sections, which occurs only when the size of the target is larger than the wavelength. In the multistatic configuration of a FSR, a certain number of transmitting and receiving positions in the air and receiving positions on the ground must be used. The altitude of the targets must always be equal to or less than the altitude of the airborne transmitter positions, which could be placed onboard UAVs or any other type of aircraft. This kind of airborne sensor network is described here for completeness, but we do not model it in the second part of the paper.

To summarize, the highest disadvantage of active radars is the need for specially designed transmitters that can be difficult to deploy.

Next, we detail some commercial active radars. The ART Midrange 3D [12] is a high-resolution C-UAS FMCW surveillance radar. This high-performance sensor is specifically designed to detect small unmanned aerial vehicles (C-UAS) and for its use in unmanned aircraft traffic management (UTM). The radar is composed of a 3D multibeam antenna system and a high-power amplification stage and is capable of detecting, tracking, and classifying micro quadcopters and micro fixed-wing UAVs, with extended elevation coverage. The main specifications of this solution can be seen in Table 1. 

Another commercial solution, provided by Indra [13], called ARMS, includes another FMCW radar. Its main characteristics are detailed next, in Table 2.

German company HENSOLDT has developed a drone detection system called Xpeller Counter UAV solution [14]. This solution can detect the potential threat through a radar system whose specifications can be seen in Table 3 (two different radar systems may be integrated).

Meanwhile, Echodyne [15] has developed an alternative active radar solution based on an Electronic Scan Antenna capable of simultaneously tracking (with very high detection rate) and searching for additional targets in its coverage. Its specifications are detailed in Table 4.

An alternative solution is the Ranger R8SS-3D from Flir [16], whose specifications can be seen in Table 5.

RST enterprise has another radar solution to detect UAVs, and it is called Doruk: UAV detection radar [17]. Its basic functions are a low-altitude moving target detection over land and sea. It provides detection, classification, azimuth and range measures, RCS, radial velocity, heading and width of Doppler Frequency Spectrum of targets. Its main specifications can be seen in Table 6.

### 2.2. Passive Detection Radars

Passive radars do not require a specially designed transmitter. There are two types of passive radar, the single station passive radar, which exploits only one illumination source, and the distributed passive radar, which uses the existing telecommunications infrastructures as illumination sources to enhance the UAV detection. Typically, two different widespread signals are used: cellular systems and the digital video broadcasting systems.

Passive bistatic radars (PBR) have a challenging problem in the detection of UAVs due to their low RCS [18]. Range migration (RM) occurs in the coherent processing interval, which makes it difficult to increase coherent integration gain and improve radar detection ability, although there are techniques to alleviate this problem. An example of single-station passive radar is the investigation presented in [19], where it is possible to localize small UAVs in 3D by exploiting a passive radar based on Wi-Fi transmissions. A demonstration of the capability of the radar to estimate the position of the target from the ground by exploiting multiple surveillance antennas is performed.

In the case of distributed passive radar, a possible approach is the one proposed in [20], where the detection system uses reflected global system for mobile communications (GSM) signals to locate and track UAVs. Another example of distributed passive radar is the one presented in [21], where a fixed-wing micro-UAV using passive radar based on digital technology is detected using audio broadcasting signals up to a distance of 1.2 km. The experiment was achieved at a lower frequency of 189 MHz in the VHF band.

The major disadvantage of passive radar is that a large amount of postprocessing effort or multiple receivers are required to obtain acceptable detection accuracy.

### 2.3. Detection through UAS Radio Frequency Signals

UAVs usually have at least one RF communication data link to their remote controller to either receive control commands (typically at 2.4 GHz) or deliver aerial images. In this case, the spectral patterns of such transmission are used for the detection and localization of UAVs. In most cases, software-defined radio receivers are employed to intercept the RF channels.

To utilize the spectrum patterns of UAVs, three possible approaches are considered for drone detection in [22]. One of them is based on sniffing the communication between drone and its controller is a clear application of this approach. Another approach is the one explained in [23], where the frequency hopping spread spectrum signals from a UAV are extracted. According to these articles, it is possible to train a classifier for identifying unique RF transmission patterns from UAVs.

Data traffic patterns are also an important feature to classify and identify UAVs. In [24], a UAV’s detection and identification system, using two receiver units for recording the received signal strength resulting from the UAV was proposed. The system makes use of a novel machine learning-based for efficient identification and detection of UAVs. The system consists of four classifiers working in a hierarchical way. In the first classifier, the availability of the sample as UAV is checked, while the second classifier specifies the type of the detected UAV. The third and fourth classifiers handle specific vendors’ drone types. The system detects UAVs flying within the area, and it can classify UAVs and flight modes of the detected UAV with an accuracy around 99%.

Another UAV detection and identification approach is based on Wi-Fi signal and radio fingerprint, as presented in [25]. Firstly, the system detects the presence of a UAV, and features from RF signal are extracted using Machine Learning and Principal Component Analysis-derived techniques to extract RF fingerprints. The extracted UAV fingerprints are stored and used as training data and test data. The results of this approach are above 95% in indoor scenarios and above 93% in outdoor scenarios.

The real scenarios are not controlled, so it is not so easy to pick up the RF signals, as there is interference in the environment. The following two studies have carried out their experiments with interference in the radio frequency band. The proposed method in [26] relies on machine learning-based RF recognition and considers that the bandwidth of the video signal and Wi-Fi are identical. The process consists of extracting 31 features from the Wi-Fi signal and the UAV video signal and then introducing them to the classifier. It is demonstrated that the proposed method can accurately recognize UAV video signal in the presence of Wi-Fi interference. The proposed method has a recognition rate greater than 95% in the 2 km outdoor experiment. On the other hand, a radio frequency-based drone detection and identification system under wireless interference (Wi-Fi and Bluetooth), by using machine learning algorithms and a pretrained convolutional neural network-based algorithm called SqueezeNet as classifiers is explained in [27]. Different categories of wavelet transforms are used to extract features from the signals. From these extracted features, different models have been built. The experiment has consisted of the study of the performance of these models under different signal-to-noise ratio levels. The results had a correct detection accuracy obtained of 98.9% at 10 dB signal-to-noise ratio level.

Next, we detail some commercial RF detection systems. DJI has created a system to detect their own drones. AeroScope [28] can identify them by monitoring and analyzing their electronic signal to gain critical information such as flight status, paths, and other information in real time. There are two types of AeroScope systems: stationary (designed for continuous protection of large-scale sites, up to 50 km range) and portable (designed for temporary events and mobile deployments, up to 5 km range).

Dedrone provides a complete airspace security system [29], including RF sensors, able to detect and localize drones by their RF signals. There are two types of these sensors: the DedroneSensor RF-160 forms the basis of the sensor network and is used in initial risk analysis, whereas the DedroneSensor RF-360 can locate and track drones. The main characteristics of these sensors can be seen in Table 7.

Finally, DroneShield provides the DroneSentry-X product [30], which is a portable device that is compatible with vehicles. It provides 360° awareness and protection using integrated sensors to detect and disrupt UAVs moving at any speed. It has a nominal UAV detection range greater than 2 km, and it detects UAV RF signals, operating on consumer and commercial industrial, scientific, and medical (ISM) frequencies.

### 2.4. Detection by Acoustic Signals

An array of acoustic sensors can be employed to capture the sound, detect, and estimate the direction of arrival of sounds from sources such as UAVs. These arrays are deployed around the restricted areas and record the audio signal periodically and deliver this signal to the ground stations. The ground stations extract the features of this audio signal to determine the direction of arrival of the UAV.

Conventionally, once the audio signal of UAV is received, the power or frequency spectrum is analyzed to identify the UAV. An example implementation of this type of UAV detection is explained in [31]. This paper shows how to estimate and track the location of a target by triangularization with two or more microphone arrays, in addition to how the UAV model can be obtained by measuring the sound spectrum of the target. In this report, a small tetrahedral array of microphones was used. The results show that the detection algorithm performs best with a 99.5% probability of detection and a 3% false alarm rate. On the other hand, the tracking algorithm often misses trajectories when other trajectories are present, and the elevation tracking is poor.

Another example of UAV detection using acoustic signals is shown in [32]. In this work, the data collection equipment is composed of two individual microphone arrays in 16-X and 4-L configurations where the microphones are placed on the ground and mounted on metal spikes, while the elevated sensors are placed on tripods. These microphones are covered by six-inch-thick foam shields to protect them and limit the effects of wind. Once the signals have been captured by the arrays, they must be processed and analyzed. The data processing developed, as well as the analysis of the acoustic sensor arrays, has been tested by being used to detect and track the trajectory of UAVs at low altitude and tactical distances. This process operates best under benign daytime conditions and is approximately five times better at detecting noisier, medium-sized, gasoline-powered UAVs than small, electric-powered UAVs.

In the literature, there are some machine learning (ML) approaches to classify the UAV from audio data. Support vector machine (SVM) is implemented to analyze the signal of an UAV engine and to build the signal fingerprint of UAV. The results show that the classifier can precisely distinguish the UAVs in some scenarios [33]. Another example of using deep learning methods to detect UAVs with acoustic signals is shown in [34]. In this paper, there is a comparison among Convolutional Neural Networks (CNNs), Recurrent Neural Networks (RNNs) and Convolutional Recurrent Neural Networks (CRNNs) using melspectrogram features. Here, the CNNs show the better performing results, achieving the highest average accuracy of 94.7%. In summary, machine learning presents an ability to recognize and locate the UAV. However, the nature of acoustic approaches limits the deployment and detection of UAV. 

In [35], a detailed study was conducted on how drone detection is performed by using acoustic signals, and it characterized how the microphone array in charge of capturing the sound signal should be organized. The geometry of the microphone array depends on the application to be carried out, although, when the desired signal can come from any angle, the best geometry is the circular array. The possible geometries studied were uniform linear array (ULA), uniform circular array (UCA) and uniform rectangular array (URA). In the array, it is important to know the number of microphones, which usually ranges from 4 to 16 microphones (in steps of two), and the distance between sensors, which usually ranges from 0.3 to 0.6 meters in increments of 0.05 meters.

A commercial C-UAS solution from Dedrone enterprise is Dedrone DroneTracker [36], which is a multiple-sensor unit that may integrate an ultrasonic audio detector. Its specifications are shown in Table 8.

### 2.5. Detection through Video/Images

Vision-based UAV detection techniques mainly focus on image processing. Cameras and videos are used to capture the images of UAVs. Then, using artificial vision techniques, UAVs positions are estimated.

A vision-based UAV detection approach is presented in [37]. This approach consists of an online recognition system for the identification of 3D objects. The system uses a black-and-white television camera to provide a 2D image on a digital computer. After obtaining the image on the computer, the next step is to remove the clutter from the image by means of a preprocess that provides a clean silhouette as well as its boundaries. At the time of the calculations, certain characteristics are obtained and are used to identify the objects, the position they occupy and their orientation in space by means of a recognition algorithm. A similar system is the one developed in [38], which makes use of classical vision algorithms. This system starts by taking the first image, which is used for initialization of the background estimation. Then a loop is started where the trajectories are predicted in the capture time for each new image taken by the cameras. All those pixels that are different from the background that was previously estimated are detected and form one or more blobs related to the current targets. These blobs are extracted using trajectory predictions, edge detectors and motion detectors. With blobs and an association process, one on more blobs are associated to each target, and in addition, the blobs within the association are used to initialize the tracks. Finally, each track is updated with its corresponding blobs, and the not-updated tracks are deleted.

In contrast, nonconventional segmentation methods make use of neural networks to directly identify the appearance of UAVs. For example, in [39], the authors developed a system that is capable of detecting, recognizing, and tracking a UAV using a single camera automatically. For that purpose, a single Pan–Tilt–Zoom (PTZ) camera detects flying objects and obtains their tracks; once a track is identified as a UAV, it locks the PTZ control system to capture the detailed image of the target region. Afterward, the images can be classified into the UAV and interference classes (such as birds) by a convolution neural network classifier trained with an image dataset. The identification accuracy of track and image reaches 99.50% and 99.89%, respectively. This system could be applied in a complex environment where many birds and UAVs appear simultaneously.

It is possible to detect UAVs from the cameras of other UAVs. An approach for online detection of small UAVs and estimation of their positions and velocities in a 3D environment from a single moving (on-board) camera is presented in [40]. The methods used are computationally light, despite the complexity of computer vision algorithms, so they may be used on UAVs with limited payload. This approach incorporates fast object detection using an AdaBoost-based tracking algorithm. Real-time performance with accurate object detection and tracking is possible, enabling the tracker to extract the position and size of an aircraft from a video frame. The detections are given to a multitarget tracker to estimate the aircraft’s position and velocity in 3D. The effectiveness of this method has been proven with an indoor experiment with three quadrotors. In [41], a general architecture for a highly accurate and computationally efficient UAV-to-UAV detection and tracking algorithm from a camera mounted on a moving UAV platform was developed. The system is composed of a moving target detector followed by a target tracker. The moving target detector accurately subtracts the background from subsequent frames by using a sparsely estimated global perspective transform. The target tracker consists of a Kalmar tracker and was validated using public video data from multiple fixed-wing UAVs working in real time. Video surveillance has not yet been incorporated to our simulation models but is described here for completeness.

Next, we describe two commercial PTZ cameras used for drone detection and tracking. On the one hand, there is Axis Q6215-LE PTZ Network Camera from Axis Communications [42], which is a camera with normal range. Its specifications can be seen in Table 9.

On the other hand, there is Triton PT-Series HD Camera from FLIR Enterprise [43], which is a PTZ with very high range, whose specifications are detailed in Table 10.

Indra also has a camera/optronic sensor to be integrated in its ARMS system. Some details on it are described next, in Table 11.

Another company that markets this type of sensor is HGH USA, specifically with its product called Spynel Series [44]. Spynel is based on thermal imaging technology with a 360° thermal sensor, which works day and night. Spynel can track targets over a long range and wide area. The specifications of each sensor model that exists in this product series can be seen in Table 12.

### 2.6. Detection by Data Fusion

Detection using a collection of these techniques is the ultimate way to detect UAVs. Data fusion, which is the process of integrating multiple data sources to obtain more consistent, accurate and useful information than that provided by any of the individual techniques explained below, has the advantaged to gain more informative and synthetic fused data than the original inputs. In the case of UAV detection, data fusion could be used to improve the performance of the UAV detection system, by overcoming or alleviating the problems and disadvantages of the individual sensors.

However, data fusion should be conducted with great caution. The key problems to be solved can be referred to as data association, positional estimation, and temporal synchronization. Data association is a general method of combining data from different sensors by correlating one sensor observation with the other observations. This process should ensure that only measurements that refer to the same drone are associated. There are different ways to perform this process: one of them is by spatial synchronization, i.e., seeing that a pair of measurements from different sensors have very similar position values. The coordinate’s changes, bias estimation and correction are sources of errors to be considered in this process. Furthermore, before making any kind of association, it is necessary to make a time synchronization so that all the measures refer to the same instant of time. The last problem faced by data fusion systems is filtering and prediction, for which they usually use common techniques such as Kalman filtering and Bayesian methods.

A low-cost, low-power methodology consisting of a fusion of technologies linking several sensors is presented in [45]. This technology includes a simple radar, an acoustic array of microphones and optical cameras that are used to detect, track, and discriminate potential airborne targets. The multimode sensor fusion algorithms employ the Kalman filter for target tracking, and an acoustic and visual recognition algorithm is implemented to classify targets. The first element of the multimode sensor network is the radar, which is responsible for detecting targets that are approaching the area of interest. The second component is the acoustic microphone array, whose main objectives are to provide target arrival direction and target identification and classification and to mitigate false alarms. The last sensor is the optical system composed of infrared detectors to improve the resolution of targets. Results show that this sensor fusion is useful for detecting, tracking, and discriminating small UAVs. Another set of heterogeneous sensors combined with a sensor data fusion is proposed in [46]. This system is composed of a Radio Frequency (RF) sensor to capture the uplink and downlink communications of the UAV, an acoustic sensor searching for the rotor noise, a passive radar system using the cellular network and a multihypothesis tracking (MHT) system for the fusion of sensor data. Finally, in the case explained in [47], the system is composed of different range acoustic, optical and radar sensors. There is a combination of sensors of long- and short-range detection, the passive RF receivers detect the UAV’s telemetry signals, and the camera and microphone sensors are used to increase the detection accuracy in the near field. Specifically, the system is composed of a 120-node acoustic array that uses acoustic signal to locate and track the UAV; 16 high-resolution optical cameras, which are used to detect the UAV in the middle distance; and MIMO radar (with three bands) to achieve remote detection in the long distance. The developed combination overcomes the drawbacks of each of the sensor types in UAV detection and maximizes the advantages of the sensors. At the same time, the system reduces the cost of large-scale sensor deployment.

In this paper, we focus on the simulation of individual sensors, so we do not simulate these integrated solutions, which remains an area for future research, especially for the cases in which some of the sensors are controlled by the outputs provided by others.

Regarding commercial solutions, some of them are based on integrating some of the previously described sensors. For instance, a commercial solution provided by Indra [13], called ARMS (Anti-RPAS Multisensor System), is a multilayer system ready to support the full C-UAS cycle, combining multiple types of sensors and countermeasures, ready to be deployed in different formats (fixed, mobile, portable) and designed to interact with complementary systems in to provide defense against UAVs threats. It is composed of a radar (described in Section 2.1), a jammer (to interfere with drone control or GPS navigation) and optronics (described in Section 2.5).

HENSOLDT Xpeller Counter UAV solution [14] combines various types of sensors and effectors for protection against small drones. The sensors used to detect and identify are radars, electro-optics, rangefinders, and direction finders. Its radars were described in Section 2.1), and it also identifies the potential threats via visual confirmation with a multispectral camera. 

Meanwhile, Dedrone provides a complete airspace security system [29]. Different types of sensors may be connected to the DedroneTracker software. The sensors provided by Dedrone are RF sensors, radars, and cameras. Depending on the application, Dedrone has different radars [48] with different performances in the Dedrone platform, such as the Counter-Drone Radar from Echodyne [15] and the Ranger R8SS-3D from Flir [16], whose specifications were analyzed in Section 2.1. The last sensors integrated by Dedrone are PTZ cameras [49]. DedroneTracker system software has a video analysis capability, able to detect and locate UAVs in real time. Depending on the application, Dedrone can integrate one or more PTZ camera models with different performance levels. On the one hand, there is Axis Q6215-LE PTZ Network Camera from Axis Communications [42], On the other hand, there is Triton PT-Series HD Camera from FLIR [43]. They were described in Section 2.5.

Another company to have its drone detection solutions analyzed in this paper is DroneShield [50]. It has a range of stand-alone portable products and rapidly deployable fixed site solutions. One of the most remarkable ones is the DroneSentry product [51], which is an autonomous fixed C-UAS system that integrates DroneShield’s suite of sensors and countermeasures into a unified responsive platform. This product has as its primary detection method the RadarZero product [52], which is a radar, and/or the RfOne RF detector [53]. It has secondary detection methods such as the WideAlert acoustic sensors and DroneOpt camera sensor [54]. The main specifications of DroneSentry can be seen in Table 13.

### 2.7. Comparative Analysis of UAV Sensing Technologies

Next, we summarize the main properties of the described technologies to summarize the previous sections. The summary takes the form of Table 14.

## 3. Counter-UAS Sensors Modeling

**Figure 1:**
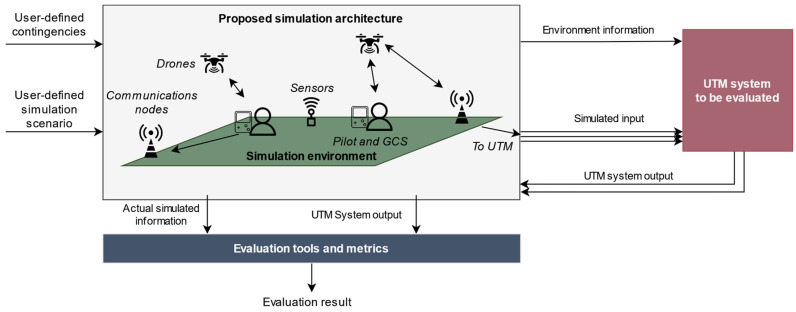
Operation of the simulation architecture proposed in [3]. Extracted from [3], with permission.

Modeling and simulation tools are a useful alternative to test and assess the performance of complex systems in a cost-effective manner. Regarding the usage of such tools to evaluate UTM systems, authors have already proposed in [3] a simulation platform that aims to replicate drone operations and complex scenarios. The objective of the platform is to easily perform system-level evaluations of UTM. To do so, the platform simulates the required input information for UTM systems both in preflight (operation definition submission for authorization) and in-flight phases (telemetry messages from drones or tracks from surveillance networks). Thus, starting from a user-defined simulation scenario (which might include the occurrence of unexpected events or contingencies), the platform is able to replicate the behavior of the actors involved in a drone operation. Then, it forwards the required data streams to the UTM system under evaluation and can retrieve the resulting output information to carry out tests and generate evaluation metrics. This operation is schematically represented in Figure 1.

The platform follows an agent modeling approach where the behavior of drones, ground control stations, surveillance networks and communication networks linking all agents is individually modeled. The complete behavior of the overall scenario arises from the autonomous interaction of these individually modeled agents. The environment in which drones operate is also simulated including terrain, weather, or airspace constraints. With this approach, the platform can currently simulate drone trajectories or effects such as navigation errors, communication disturbances (i.e., latencies, package losses…) and drone detection from sensors.

**Figure 2:**
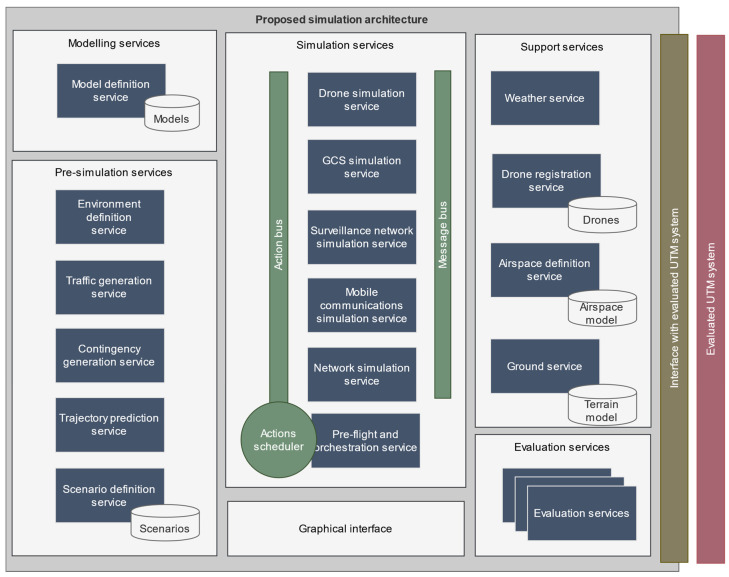
Detail of the microservice-based, extendable simulation architecture proposed in [3]. Extracted from [3],.with permission.

A model-agnostic, extendable microservices-based architecture has been used to implement the platform, as depicted in Figure 2. The architecture allows for defining multiple simulation models for each agent that can be easily implemented and simultaneously simulated. The simulation of each agent is isolated within a separate microservice so that modeling changes in each service do not affect the rest of the platform. It also provides utilities to define replicable simulation scenarios where the simulated agent’s specification can be defined together with the selected model to carry out their simulation. 

A set of simple simulation models for each agent was initially provided, as described in [3]. Particularly, a simplistic technology-agnostic model for noncooperative sensors was already provided. This model just considered a maximum range for each sensor following a pass–not pass approach. It also included a constant additive gaussian noise to model detection inaccuracies. 

The models proposed in this section for different technologies aim to improve that simplistic model by designing more accurate models that are based on the inner operation of each sensor type. Measurement simulation models are proposed in this paper for the following sensors: active radars, passive radars, and microphone sensors.

By integrating these enhanced models into the existing platform (which can be easily done by modifying the preexisting surveillance network simulation service), it is possible to assess the performance of those sensors in realistic scenarios. Simulation scenarios defined for the platform not only consider the number of drones, their trajectories and the distribution of surveillance sensors; they also allow for simulating emergent effects from the interaction of sensors with other agents. For instance, the simulator is also able to simulate the network used by sensors to forward information to a UTM system and how it affects track reporting periodicity, latencies, etc. To summarize, the models proposed in this section will enhance the capabilities of the preexisting simulation tool, but they will also benefit from the integration in such platform for assessing the performance of surveillance sensors.

### 3.1. Active Radar

Two different types of active radars have been modeled, quasi-monostatic radars and MIMO radars.

#### 3.1.1. Quasi-Monostatic Radars

This radar will be simulated using a power model from the radar equation. It will be assumed that the separation between transmitter and receiver is small compared to the distance to the target. In this first approximation, it is assumed that the radar can eliminate the clutter by doppler filtering. It is also assumed that the predominant noise is thermal. Its calculation now depends on the surrounding conditions and not only on the bandwidth. The main characteristics are:

- Radar cross section dependent on target size [55].
- CFAR detection.
- Radar parameters adapted to drone detection (integration times of the order of tens of milliseconds and range resolutions in order of meters).
- Exploration times around a second.
- Measurement error simulation.
- Minimum scan time below a second.
- False alarm simulation.

The basic parameters defining the model of a quasi-monostatic radar are:

- Instrumental range ($R_{max}$).
- Minimum and maximum azimuth of coverage.
- Distance resolution in meters ($\Delta_{dis}$).
- Bandwidth.
- Transmitting array position.
- Transmitted power in W.
- Azimuthal width of the transmission pattern ($\theta_{3dBaz-T}$).
- Elevation width of the transmission pattern ($\theta_{3dBele-T}$).
- Position of the receiving array.
- Number of receiver array beams ($N_{beams}$).
- Receiver array azimuth beamwidth ($\theta_{3dBaz-R}$).
- Receiver array elevation beamwidth ($\theta_{3dBele-R}$).
- Dwell time.
- Minimum time between scans.
- Minimum and maximum frequency.
- False alarm probability.

The typical expression for the radar equation of a quasi-monostatic radar is simpler than that of a microwave radar since the free-space propagation losses are included within the ground-wave propagation losses. The radar equation is:

(1) $$\frac{S}{N}=P_{av}\frac{G_{T}G_{R}\sigma \lambda^{2}\;T_{int}}{{\left(4\pi \right)}^{3}\;R_{T-t}^{2}R_{t-R}^{2}\;L_{s}\;N_{0}}$$

where:

- $\frac{S}{N}$ signal noise relation in the detector
- $P_{av}$ average power of the system
- $G_{T}$ transmit antenna power gain
- $G_{R}$ receiver antenna power gain
- σ cross section
- λ wavelength
- $T_{int}$ integration time
- $R_{T-t}$ transmitter–target distance
- $R_{t-R}$ target–receiver distance
- $L_{s}$ power losses of the radar system
- $N_{0}$ system noise

To obtain the elevation gain, the elevation width is considered, and to obtain the antenna gain, the azimuth shaping is considered. In this case and considering that the beamforming is conducted only in azimuth, the array gain is estimated approximately as 360° divided by the beamwidth.

(2) $$G_{T}≅\left(\frac{4\pi }{\theta_{3dBele-T}\theta_{3dBazi-T}}\right)$$

(3) $$G_{R}≅\left(\frac{4\pi }{\theta_{3dBele-R}\theta_{3dBazi-R}}\right)$$

(4) $$\theta_{3dB-R}=\frac{\theta_{max}-\theta_{min}}{N_{beams}}$$

The system losses depend on many factors such as the antenna feed or the construction of the processing. A loss factor of around 4 dB has been given as a typical value. The cross section for these frequency bands depends on the target size, and it is modeled as constant for all angles (0.01–0.1 m^2^).

The reception noise is predominantly thermal noise due to the frequencies being used.

(5) $$N_{0}=k\;T_{0}\;10^{F_{a}/10}$$

where *k* is the Boltzmann constant, $T_{0}$ is the Earth temperature (typically 290° K) and $F_{a}$ a noise factor with a typical value of 5 dB.

Once the SNR has been calculated, the detection, false alarms and measurement position must be generated. The detector is assumed to be a CA-CFAR so it is assumed that the target behaves as a Swerling I between scans and the noise residual has a Gaussian distribution [56]. The threshold of the CFAR is obtained with the following expression:

(6) $$\alpha ={\left(PFA\right)}^{-1/N}-1$$

where *α* is the CFAR threshold factor, *N* is the number of CFAR cells and *PFA* is the false alarm probability.

The probability of detection (*PD*) is calculated according to the following expression corresponding to a CA-CFAR and a Swerling I target in Gaussian noise:

(7) $$PD={\left(1+\frac{\alpha }{\left(1+\frac{S}{N}\right)}\right)}^{-N}$$

The generation of whether there is detection or not is completed by generating a uniform random variable and comparing it with the probability of detection:

(8) Detection=(rand(0,1)≤PD)

On the other hand, several false alarms per lap will be generated and output at each scan of the space. The average number of alarms per lap is calculated with the following expression:

(9) $$N_{alarms}=PFA\;\left(\frac{R_{max}}{\Delta_{dis}}\right)N_{beams}$$

A binomial random variable with mean $N_{alarms}$ is generated for each scan. The positions corresponding to the N false alarms are then generated uniformly in azimuth, elevation and distance. The position of each alarm is generated as:

(10) $$\rho_{i}=R_{max}\;Rand\left(0,1\right)$$

(11) $$\theta_{i}=\theta_{min}+\left(\theta_{max}-\theta_{min}\right)\;Rand\left(0,1\right)$$

(12) $$h_{i}=20+100\cdot \;Rand\left(0,1\right)$$

If there has been detection, the measurement position is calculated assuming the quasi-monostatic configuration and adding to the true position of the aircraft errors in the radial direction and tangential to the direction of view from the receiver. It is assumed that the optimal distance, elevation, and azimuth estimators are being used. The expressions of their errors are given below.

(13) $$\sigma_{d}=\frac{\Delta_{dis}}{1.63\sqrt{2\left(\frac{S}{N}\right)}}$$

(14) $$\sigma_{azi}=\frac{\theta_{3dBazi\_R}}{2\sqrt{\left(\frac{S}{N}\right)}}$$

(15) $$\sigma_{ele}=\frac{\theta_{3dBele\_R}}{2\sqrt{\left(\frac{S}{N}\right)}}$$

#### 3.1.2. MIMO Radars

This simulator models a MIMO radar with spatially separated antennas at high frequencies (X, Ku, K or Ka). Each radar unit will have three transmitting antennas and one receiving antenna placed with the central transmitter. Since the antennae are widely separated and will view the target from different angles, echo coherence is not expected. Therefore, incoherent integration processing is performed, since the coherent has no gain. The main characteristics are:

- Radar cross section dependent on drone size.
- Several simultaneous transmitters.
- CFAR detection.
- Measurement error simulation.
- Simultaneous space exploration system using simultaneous antenna beams (MIMO techniques).
- Minimum scan time around one second.
- False alarm simulation.

The basic parameters defining the model of a MIMO radar are:

- Transmission frequency.
- Position of each transmitting and receiving antenna.
- Power transmitted by each transmitter.
- Instrumental range.
- Minimum azimuth of coverage.
- Maximum azimuth of coverage.
- Azimuthal width of the transmission pattern ($\theta_{3dBaz-T}$).
- Elevation width of the transmission pattern ($\theta_{3dBele-T}$).
- Number of receiver array beams ($N_{beams}$).
- Receiver array azimuth beamwidth ($\theta_{3dBaz-R}$).
- Receiver array elevation beamwidth ($\theta_{3dBele-R}$).
- Integration time.
- Bandwidth.
- Distance resolution.
- False alarm probability.
- Scan period.
- Antenna gain.
- Secondary lobe level.

In this case, to obtain the target echo power at the receiver, it has been added the echo powers of each of the three receivers. It is assumed that, in MIMO radar, before the incoherent integration of the signals from all transmitters, the possible clutter is coherently eliminated, but in this model, clutter is not considered. Therefore, the power received from a target will be obtained as the sum of the power received from each transmitter.

(16) $$P_{target}=P_{Tx1}+P_{Tx2}+P_{Tx3}$$

(17) $$P_{Txi}=P_{av\_i}\frac{G_{Txi}G_{R}\;T\;\lambda^{2}\;\sigma \;}{{\left(4\pi \right)}^{3}R_{1}{}^{2}R_{2}{}^{2}\;L_{p}}\cdot F_{p\_Txi\_Rx}$$

where:

- $P_{av\_i}$ average power of transmitter i
- $G_{Txi}$ power gain of transmitting antenna i
- $G_{R}$ receiver antenna power gain
- λ wavelength
- σ cross section
- T integration time
- *R_i_* distance in each path
- $L_{p}$ power losses
- $F_{p\_Txi\_Rx}$ propagation factor in the propagation path ith-transmitter–target–receiver

The average signal-to-noise ratio per echo for each transmitter is calculated by adding the target powers from each transmitter and dividing by the number of transmitters and dividing by the noise power.

(18) $$\left(\frac{S}{N}\right)=\frac{P_{target}/N}{P_{N\_elec}}$$

Transmitting antennas are assumed to be uniformly patterned in coverage in the horizontal plane and to distribute their power to uniformly illuminate the scanned area from their respective positions. Transmitter gains are specified as a factor depending on the azimuth width of the scanned area. The gains are as follows:

(19) $$G_{T}≅\left(\frac{4\pi }{\theta_{3dBele-T}\theta_{3dBazi-T}}\right)$$

(20) $$G_{R}≅\left(\frac{4\pi }{\theta_{3dBele-R}\theta_{3dBazi-R}}\right)$$

The propagation factor, being free space, is assumed to be 1. The system losses are assumed to be 2 dB due to Doppler filtering envelopes and CFAR detection losses. Assuming that the images of the three transmitters are integrated incoherently, it can be assumed that it is integrated a CFAR reference of three times the reference of a single system. If it is assumed for each radar a 10-cell reference, it will have 30 reference cells. For a PFA of 1e-4, this means a loss of less than 1 dB [57]. The cross section for these radars depends on the target size, so it is specified by an equal constant for all angles (0.01–0.1 m^2^).

These radars operate at high microwave frequencies, and consequently, the predominant noise is thermal noise. Therefore, the noise power is obtained as:

(21) $$P_{N\_elec}=k\;T_{0}f_{N\_total}$$

where $f_{N\_total}$ is the receiver and antenna noise figure (it has been taken a typical value of 5 dB). The bandwidth is not shown since it is assumed that the receiver uses a matched filter, and for the calculation of the ($\frac{S}{N}$) ratio, the integration time has already been included in the radar equation.

The detection will be calculated after integrating the echoes from the three transmitters in an incoherent way by making the appropriate corrections according to the relative positions of the transmitters. After filtering, the square of the envelope is found, and the one coming from the three transmitters is integrated. The expression of the detection threshold is obtained according to the following expression [58]:

(22) $$PFA=1-P\left(Y_{b},N\right)$$

where $P\left(Y_{b},N\right)$ represents the incomplete gamma function of order *N* (the number of transmitters), and $Y_{b}$ is the detection threshold for the specified PFA. The threshold is calculated by clearing the above equation using the inverse function of the incomplete gamma function. The probability of detection (*PD*) is calculated according to the following expression, corresponding to the integration of the echo from all transmitters:

(23) $$PD=1-P\left(\frac{Y_{b}}{\left(1+\left(\frac{S}{N}\right)/2\right)},N\right)$$

where $\frac{S}{N}$ is the average signal-to-noise ratio per transmitter, calculated with the radar equation. These expressions are for the integrator without CFAR. The effect of CFAR has been included through a term in the power losses. The generation of whether there is detection or not is decided by generating a uniform random variable and comparing it with the probability of detection as in the quasi-monostatic radar.

On the other hand, a few false alarms per lap and their positions will be generated as in the quasi-monostatic radar. Finally, if there has been detection, the measurement position is also calculated as in the quasi-monostatic radar.

### 3.2. Passive Radar

Passive radars will be simulated using a power model from the radar equation, which includes a multipath propagation model. These radars are of multistatic type. The predominant noise will be the direct transmitter–receiver signal interference, which will be considered in the model. The main characteristics are:

- Radar cross section dependent on drone size.
- Possibility of several simultaneous opportunity transmitters.
- Opportunity transmitters self-interference simulation.
- Simulation of electrical noise dependent on the frequency of the transmitter (atmospheric, human and white noise).
- CFAR detection.
- Measurement error simulation.
- Simultaneous space exploration system using multilateration position determination techniques (bistatic or multistatic radars).
- Minimum scan time in the order of seconds (>1 s).
- False alarm simulation.

The basic parameters defining the model of a passive radar are:

- Passive radar position.
- Instrumental range in kilometers.
- Minimum and maximum azimuth of coverage in degrees.
- Number of receiver antenna beams.
- Number of opportunity transmitters.
- Position of each opportunity transmitter.
- Carrier frequency of each opportunity transmitter.
- Transmitted power of each opportunity transmitter.
- Antenna gain.
- Gain of the receiving antenna of the direct signal.
- Bandwidth of each opportunity transmitter.
- Integration time in seconds.
- Number of receiver array beams ($N_{beams}$).
- Receiver array azimuth beamwidth ($\theta_{3dBaz-R}$).
- Receiver array elevation beamwidth ($\theta_{3dBele-R}$).
- Receiving array sidelobe level.
- Direct signal cancellation level
- False alarm probability.
- Scan period in seconds.

The radar equation for a passive radar is implemented in several steps. The first is to calculate the received signal power of the target echo before the correlator for an opportunity transmitter.

(24) $$P_{target}=P_{av}\frac{G_{T}G_{R}\;\lambda^{2}\;\sigma_{final}\;}{{\left(4\pi \right)}^{3}R_{1}{}^{2}R_{2}{}^{2}\;L_{p}}\cdot F_{p}$$

where:

- $P_{av}$ average power of transmitter
- $G_{T}$ transmitting antenna power gain
- $G_{R}$ receiver antenna power gain
- λ wavelength
- σ cross section
- *R_i_* distance in each path
- $L_{p}$ power losses
- $F_{p}$ propagation factor

The ratio ($\frac{S}{N}$) is calculated at the correlator output, where the signal will have a gain equal to the square of the product bandwidth and propagation time. The interference powers (noise and correlator side lobes of different signals, including the target) have a gain equal to the product of bandwidth and integration time. The $\frac{S}{N}$ ratio to be used in the CFAR is computed with the following expression:

(25) $$\left(\frac{S}{N}\right)=\frac{P_{target}\cdot {\left(B\;T\right)}^{2}}{P_{target}\cdot \left(B\;T\right)+P_{N\_clutter}\cdot \left(B\;T\right)+P_{N\_signal}\cdot \left(B\;T\right)+P_{N\_elec}\cdot \left(B\;T\right)}$$

where $P_{N\_clutter}$, $P_{N\_signal}$ and $P_{N\_elec}$ are the clutter, direct signal and electrical noise powers at the correlator input, respectively (these are calculated in the corresponding sections); *T* is the integration interval and *B* is the bandwidth (these parameters are specified for each opportunity signal).

Transmitting antennas are assumed to have uniform pattern in coverage in the horizontal plane. The transmit gain is specified as a parameter of the transmitter. This gain, if omnidirectional broadcasting is assumed, will be around that of a half-wave dipole (2.15 dBi). As the transmitter power is usually given in apparent radiated power, which considers the gain of the transmitting antenna over the half-wave dipole, 2.15 dBi is the gain over the isotropic that we will assume of the transmitting antenna. For the receiving antenna, a circular array is assumed that generates a number N of beams covering 360°.

(26) $$G_{T}=10^{G_{T}/10}$$

(27) $$G_{R}≅\left(\frac{4\pi }{\theta_{3dBele-R}\theta_{3dBazi-R}}\right)$$

The above gain expression will be used for signals within the main beam. For signals entering through the secondary lobes (in the case of passive radars, it will consider the direct signal entering through the secondary lobes of the antenna), it is considered that the signal suffers a constant gain equal to the level of the secondary lobes of the antenna.

(28) $$G_{R\_sidelobes}=\left(\frac{4\pi }{\theta_{3dBele-R}\theta_{3dBazi-R}}\right)\cdot \;10^{G_{r\_sidelobe}/10}$$

The propagation factor, being free space, is assumed to be 1. System losses, for passive radars, are 2 dB due to the correlator windowing to reduce the secondary lobes in distance and doppler and another 2 dB due to the clutter elimination system and direct signal. The cross section for these radars depends on the target size, so it is specified by an equal constant for all angles (0.01–0.1 m^2^). 

The noise of a passive radar is composed of three main components: the radio noise; the self-interference of the signal itself due to the secondary lobes of the cross-correlation function; and the residual of the secondary lobes of the direct signal autocovariance function. In this model, clutter power is assumed to be zero.

The power of the direct signal arriving at the receiver is calculated using the propagation equation and applying the attenuation that a typical direct signal canceller can provide.

(29) $$P_{N\_signal}=P_{av}\frac{G_{T}\;G_{R\_sidelobe}\;\lambda^{2}}{{\left(4\pi \right)}^{2}R_{b}{}^{2}}\cdot L_{direct}$$

After the correlator, the echo of the direct signal appears at zero distance and is eliminated. What remains is the residue of the secondary lobes of the ambiguity function spread over the entire Doppler–distance space.

The reception noise in this type of band is the antenna noise (human noise + galactic noise + atmospheric noise). This noise predominates over the thermal noise. Finally, the power of the radio noise can be obtained as:

(30) $$P_{N\_elec}=k\;T_{0}f_{N\_total}B$$

(31) $$f_{N\_total}=10^{F_{a}/10}-1+L_{l}L_{a}f_{Rx}$$

where $F_{a}$ is calculated as in quasi-monostatic radars, $L_{l}$ represents the transmission line losses (typically 0.5 dB [57]), $L_{a}$ represents the resistive losses in the antenna (typically 0.5 dB [57]) and $f_{Rx}$ represents the receiver noise figure (typically 4 dB [57]). In this first approximation, it has been estimated that there is no clutter.

Once the signal-to-noise ratio is obtained, detection and false alarms are generated. This generation is the same as that of the quasi-monostatic radars, so the explanation of its procedure can be seen in that section except elevation since these passive radars do not calculate target height. The measured position will be generated by adding to the actual position a random variable with standard deviation the noise variance. The measurement accuracy is calculated in local radar coordinates centered on the receiver (*y*-axis north, *x*-axis east). First, the covariance matrix of the measurement is calculated in local Cartesian as [59]:

(32) $$P=\left[\begin{array}{cc} \sigma_{x}{}^{2} & \sigma_{xy}\\ \sigma_{yx} & \sigma_{y}{}^{2} \end{array}\right]=\left\{\frac{\delta \hat{x}}{\delta r_{R\theta }}\right\}\left[\begin{array}{cc} \sigma_{R}{}^{2} & 0\\ 0 & \sigma_{\theta }{}^{2} \end{array}\right]{\left\{\frac{\delta \hat{x}}{\delta r_{R\theta }}\right\}}^{T}$$

(33) $$\left\{\frac{\delta \hat{x}}{\delta r_{R\theta }}\right\}=\left[\begin{array}{cc} \frac{\delta \hat{x}}{\delta R} & \frac{\delta \hat{x}}{\delta \theta }\\ \frac{\delta \hat{y}}{\delta \theta } & \frac{\delta \hat{y}}{\delta R} \end{array}\right]$$

(34) $$\hat{x}=\frac{\left({\left(R+R_{b}\right)}^{2}-R_{b}{}^{2}\right)\;sin\left(\theta \right)}{2\left(R+R_{b}-R_{b}\;sen\left(\theta \right)\right)}\;$$

(35) $$\hat{y}=\frac{\left({\left(R+R_{b}\right)}^{2}-R_{b}{}^{2}\right)\;cos\left(\theta \right)}{2\left(R+R_{b}-R_{b}\;sen\left(\theta \right)\right)}$$

where $\sigma_{R}$ and $\sigma_{\theta }$ are the standard deviations, which are calculated as in the quasi-monostatic radar; *R* is the bistatic distance ($R_{1}+R_{2}-R_{b}$) and $R_{b}$ is the baseline distance (transmitter–receiver).

Finally, the output positions are found by generating a 2D Gaussian random variable correlated with the previous autocorrelation matrix:

(36) $$\left[\begin{array}{c} x_{measure}\\ y_{measure} \end{array}\right]=\left[\begin{array}{c} x\\ y \end{array}\right]+\left\{\frac{\delta \hat{x}}{\delta r_{R\theta }}\right\}\left[\begin{array}{c} \sigma_{R}\cdot \;randn\left(1\right)\\ \sigma_{\theta }\cdot \;randn\left(1\right) \end{array}\right]$$

### 3.3. Microphone Sensor and RF Sensor

Microphone sensors and RF sensors are modeled following an azimuth model in which, from the power of the signals sent by the drones, either acoustic or radio frequency, the signal-to-noise ratio at the input of the sensor is calculated, and from the detection, the azimuth and distance to the sensor are obtained. These sensors have a certain sensitivity, and, depending on the signal-to-noise ratio, there will be detection or not. The main characteristics are:

- Surface propagation losses over land.
- Parameters adapted to drone detection (integration times of the order of minutes).
- Exploration times on the order of minutes.
- Measurement error simulation.
- False alarm simulation.

The basic parameters defining the model of a sensor are:

- Sensor position.
- Sensor sensitivity.
- Distance resolution in kilometers.
- Maximum range in meters.
- Number of sensors.
- Minimum and maximum azimuth in degrees.
- Bandwidth.
- Receiving beamwidth in degrees.
- Direct signal cancellation level.
- False alarm probability.

The first is to calculate the received signal power of the target echo before the correlator.

(37) $$P_{target}=\frac{P_{average}}{D_{C}\cdot Att\cdot L_{p}}$$

where $P_{average}$ is the noise power of the drone. $D_{C}$ is the directivity correction, Att is the attenuation and $L_{p}$ is the system power loss.

The ratio ($\frac{S}{N}$) is calculated at the correlator output. There will be the interference powers of noise and correlator side lobes of different signals, including the target. The $\frac{S}{N}$ ratio is computed with the following expression:

(38) $$\left(\frac{S}{N}\right)=\frac{P_{target}}{P_{N\_clutter}+P_{N\_signal}+P_{N\_elec}}$$

In this case, a multisource scenario is assumed, so the directivity correction factor is set at 3 dB. Once the source, the powers and their basic definitions have been characterized, we proceed to obtain the calculation of the effects that produce attenuation. The attenuation in real environments for the propagation of a wave is defined by the following equation:

(39) $$Att=A_{div}+A_{atm}+A_{gr}+A_{bar}+A_{misc}$$

The waves emitted by a drone are those of an omnidirectional source, since it propagates in all possible directions, so the waves emitted are spherical waves whose power level coincides at the same distance from the source. As this distance increases, the wave energy is distributed over a larger and larger area, so that each time this distance is doubled, the power level decreases by a factor of 6 dB theoretically, so the geometric divergence attenuation is:

(40) $$A_{div}\left(\mathrm{dB}\right)=20\cdot \log_{10}\left(R\right)+11$$

where *R* is the distance in meters between the drone and the sensor.

Atmospheric absorption is the attenuation due to nitrogen, oxygen and carbon dioxide during wave propagation as it travels a specific distance to the receiver.

(41) Aatm(dB)=α(dBkm)·R(km)

where α(dBkm) is the atmospheric attenuation coefficient, which depends on the following parameters: the frequency of the wave, the ambient atmospheric temperature, the relative humidity of the air and the ambient pressure. Since there are already estimated tables from which these values can be obtained and for the frequencies of these waves, this coefficient takes values of the order of 1 × 10^−3^ to 1 × 10^−2^.

Ground attenuation is mainly due to waves reflected by the ground surface interfering with the propagation of the main wave from the source to the receiver. This attenuation occurs when the source or receiver is close to the ground surface. This model uses an equation that allows for obtaining the ground effect attenuation in a simpler way because its operation is specified only for long distances and with porous or mixed surface. As the source gets closer, this attenuation tends to disappear.

(42) $$A_{gr}\left(\mathrm{dB}\right)=4.8-\left(2\cdot \frac{h_{m}}{R}\right)\cdot \left[17+\left(\frac{300}{R}\right)\right]$$

where $h_{m}$ is the average height of the propagation path above ground in meters, and R represents the distance from the drone to the receiver, also in meters.

An object should be considered as a shielding obstacle (barrier) if: it meets a surface density of at least 10 kg/m^2^, it has a closed surface with no large cracks or gaps and the horizontal dimension of the object perpendicular to the line connecting transmitter–receiver is greater than the wavelength. As the simulator is going to operate in real spaces that are filled with objects, it is assumed that the barrier losses are 3 dB.

Finally, there may be other types of attenuation such as those due to foliage or housing. Losses of 3 dB are assumed.

The reception noise is assumed to be the microphone noise (human noise + atmospheric noise + natural interferences). This noise predominates over the thermal noise. Thus, the power of the audio noise is assumed to be a constant ($P_{N\_elec}$). Once the signal-to-noise ratio is obtained, detection is generated. Logistic regression was used to determine the probability of detection. If the signal-to-noise ratio exceeds the sensitivity of the sensor there is detection, the model used is as follows:

(43) $$PD={\frac{1}{1+e^{-\left(\left(\frac{S}{N}\right)-Sensitivity\right)}}}^{\;}$$

The generation of whether there is detection or not is confirmed by generating a uniform random variable and comparing it with the probability of detection:

(44) Detection=(rand(0,1)≤PD)

Later, false alarms generation and the generation of measurement positions is performed. These generations are the same as those of the quasi-monostatic radars, so the explanation of their procedures can be seen in that section. It should be noted only azimuth measurements are obtained (through the measurement of the angle of arrival), as range measurement from acoustic signals would demand the performance of multistatic/trilateration procedures.

## 4. Counter-UAS Simulation Results

The previous models have been implemented and integrated in the simulator described in [3]. This integration allows us to use the proposed models in realistic drone scenarios to assess and compare the performance of different sensors. Particularly, a scenario is proposed in this section where an area of interest (i.e., a critical facility) is to be surveilled with different C-UAS sensors.

**Figure 3:**
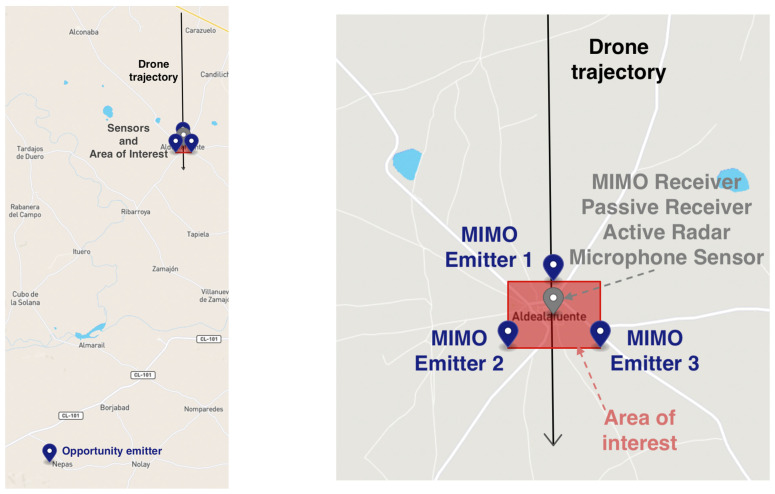
Graphical representation of the proposed scenario.

The simulated scenario is represented in Figure 3, where the area of interest to be protected is depicted in red. A surveillance solution using radars and eventual microphone sensors is proposed. This solution is complemented with the microphone sensor that works in shorter distances. The proposed sensors (depicted as markers in Figure 3) are:

- **Quasi-monostatic Radar** (also named Active Radar in figures): A quasi-monostatic radar has been installed in the middle of the critical infrastructure with a quasi-monostatic configuration. The values taken to model the sensor refer to some of the commercial radars detailed in the state-of-the-art section. The radar has an instrument range of 10 km, a minimum azimuth of −180° and a maximum of 180°, 32 receiving beams and 10 m resolution. The average power transmitted is 500 W. The minimum time between explorations is 0.06 sec (the dwell time). The minimum frequency is 8 GHz, and the maximum frequency is 12 GHz. Finally, the reception beamwidth in azimuth is 2°, and the beamwidth in elevation is 6°.
- **Passive Radar**: A passive radar is also installed in the center of the protected facility. It works in conjunction with a hypothetical transmitter of opportunity (i.e.: DVB-T transmitter) located at around 20 km from the passive receiver. The transmitter has enough power to support an instrument range of around 10 km. The minimum azimuth is −30°, and the maximum is 30°. The resolution in distance is about 20 m, and the resolution in azimuth is 2°. A scan time of 1 second has been assumed since it is a system with simultaneous space exploration without mechanical antenna movement. The antenna has a gain of 2 dBi, a secondary lobe level of 22 dB and a signal cancellation level of 60 dB. Finally, the carrier frequency is 600 MHz.
- **MIMO Radar**: A MIMO radar is also installed in the critical infrastructure. It has the following instrumental coverage (minimum azimuth: −180°, maximum azimuth: 180°, maximum range: 10 km): It is a medium/short surveillance system with one receiver (located in the center) and three transmitters (located in the area perimeter), which receive simultaneously through 32 receiving beams. The power transmission of each transmitter is 2 kW. The maximum scan rate is 1 s. The resolution in distance is about 20 m, and the resolution in azimuth is 3°. The antenna has a gain of 11.6 dBi, a secondary lobe level of 13 dB and a signal cancellation level of 40 dB. Finally, the carrier frequency is 15 GHz.
- **Microphone sensor**: A microphone sensor is also simulated in this scenario located in the center of the critical area. It is an array composed by eight microphones separated 0.5 m from each other. The sensitivity of the array is 32 dB, and it has an instrumental range of 1 km. The minimum azimuth of the sensor is −180°, and the maximum azimuth is 180°.

Although not frequent, violent and hostile acts against critical infrastructures have already occurred and are documented. The scenario tries to assess the alert distance in case of an aerial attack and the positioning accuracy provided by each of the sensors in the described surveillance system. The simulated attack is to be conducted by a terrorist group that intends to infiltrate by air using an off-the-shelf, affordable, and small drone such as a DJI Phantom 4 (estimated RCS of 0.01 m^2^ and a noise level of around 80dB). The departure place is located around 7 km away from the critical infrastructure. From there, the drone will try to make a direct approach at maximum speed following the direction depicted as a black arrow in Figure 3). 

**Figure 4:**
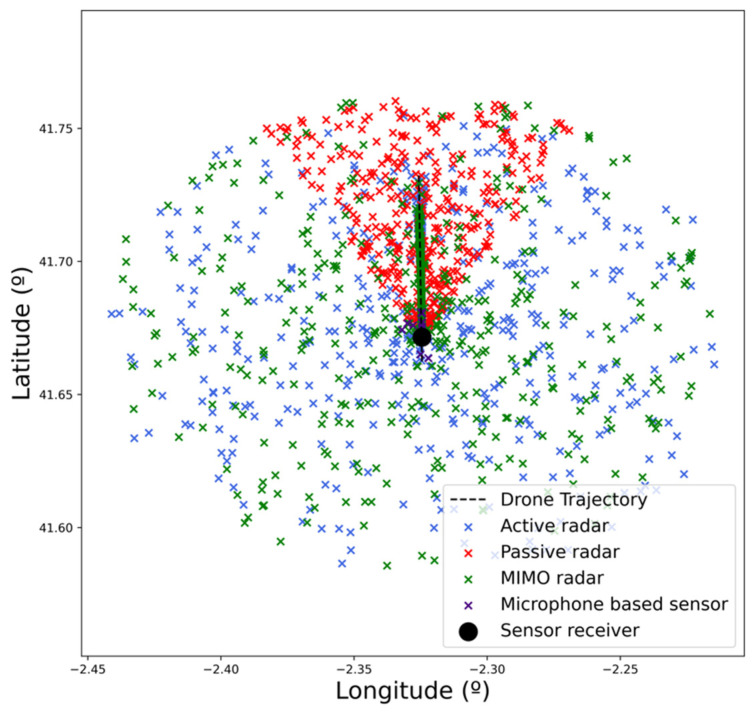
Plots generated by each of the sensors.

This scenario (i.e., drone trajectory, sensors’ location) has been easily represented and executed in real time using the simulation platform. After running it, the plots generated by each of the sensors were retrieved for analysis. These plots are shown in Figure 4, where the plots corresponding to actual drones and the false alarms are represented. To represent the plots from the microphone sensor (where only angular information is available), the actual distance of the drone is used.

**Figure 5:**
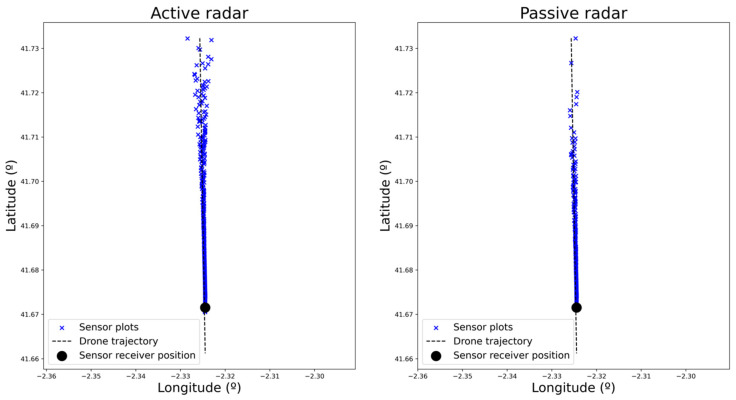
Plots generated by the active and passive radar sensors corresponding to the actual incoming drone.

**Figure 6:**
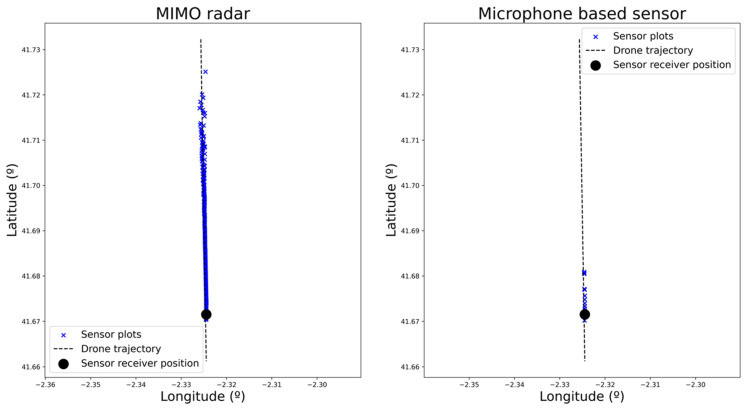
Plots generated by MIMO and microphone-based sensors corresponding to the actual incoming drone.

**Figure 7:**
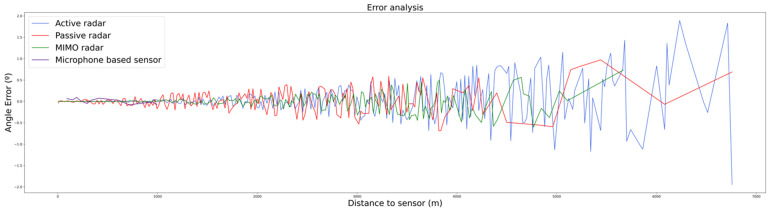
Angle error of each of the plots as distance to each of sensors’ receivers increases.

False alarms are filtered out in Figure 5 and Figure 6 for each of the four sensors to facilitate the analysis. There, the alert distance provided by each of the sensors can be compared. As expected, radar-based sensors have a greater range than the microphone-based one, which only detects the drone in very close proximity. Within the radar-based sensors, active radar provides consistent detections from the beginning of the trajectory, whereas passive and MIMO sensors provide consistent results from a range of 3 and 4 km, respectively (as can be derived from plot density in Figure 7). It can also be checked that detection probability (related to the number of detections) increases as the distance to the sensor decreases. This result is the expected one, as detection probability increases with the SNR, which also increases as the distance to the sensor is reduced.

**Figure 8:**
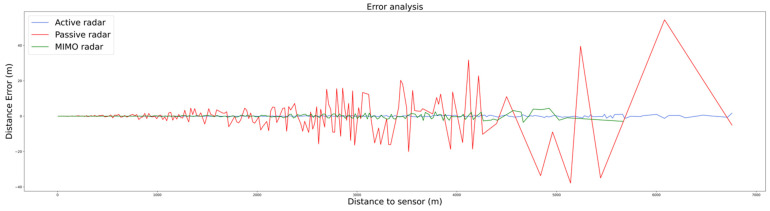
Distance error of each of the plots as distance to each of sensors’ receivers increases.

The positioning errors are depicted in Figure 7 for the angle error and Figure 8 for the distance error (microphone sensor is not included here). It can be observed that the magnitude of both type of errors decreases as the drone approaches the sensor receiving location. This can be explained, once again, by the dependency between the computed error and the SNR.

More detailed performance measures could be obtained, both in terms of detection and accuracy (i.e., PD vs. range or angle/range error standard deviations vs. range). In the case of monostatic radars/RF sensors and acoustic sensors, it is possible to derive this relation by considering PD/accuracy dependency with SNR, which, in turn, depends directly on range. However, for multistatic sensors, passive radars and distributed sensors in general, the relations are much more unlinear, and the results are very scenario dependent. In these cases, the relative locations of the emitters, receivers, etc. have important impact on the results.

## 5. Conclusions and Future Work

This paper reviews some of the current technologies used for the noncollaborative detection and tracking of UAVs and proposes a collection of simulation models, composed by integrating preexisting models of radar and acoustic sensing and by adapting them to our application. These models allow for a lightweight simulation of the most important effects on detection and estimation performance of the C-UAS sensors and sensor networks. 

There are some limitations on the current models, such as:

1. The radar models are not fully compatible with some newer Electronic Scan Antenna radars with adaptable track update rates.
2. The problem of target resolution has not been addressed, which could impose limitations to the simulation of nearby targets and drone swarms.
3. Multistatic sensors with several receivers have not been implemented.
4. Systematic errors (biases) related to sensor alignment, propagation, etc. have not been included in our models.

The presented simulation results show the capability to derive realistic measures using those simulation models, following the expected behaviors regarding both detection and estimation accuracy performance. Finally, there are a collection of future lines for research related to this paper:

1. Improvements of the models to alleviate the previous limitations.
2. Completion of the simulation with models adequate for RF UAS signal detection. The model is expected to be similar to that of the acoustic system, with specific modifications to define the noise power and the emitter signal power.
3. Completion of the simulation with models adequate for camera/vision systems.
4. Definition of simulation means to evaluate integrated deployments using different collaborating sensors and potentially managing the collection of sensors for specific tasks (long-range detection, short-term classification, clutter removal, etc.)
5. Integration with actual UTM systems and tracking systems.

## Figures and Tables

**Table 1:** ART Midrange 3D specifications.

Specification	Value
Frequency Band	Ku-band
Bandwidth	1 GHz
Elevation Control	+/−5 degrees
Instrumental Detection Range	5000 m
Coverage Area	78 km^2^
Azimuth Coverage	360°
Scan Rate	60 rpm (configurable)
Range Resolution	1 m–0.2 m
Range Accuracy	0.25 m–0.05 m
Communications	TCP/IP over Ethernet
Protocol	XML-based on NMEA0183

**Table 2:** ARMS radar specifications.

Radar
Ku-band, FMCW
Scan 360 degrees/second
Sectorized RF blanking
Doppler and Clutter Map techniques
True track report (position, course and speed) >2 km for smallest target of RCS = 0.1 m^2^, once per second
X-Band alternative for longer ranges

**Table 3:** HENSOLDT radar specifications.

Specification	Spexer 2000 3D MkII Radar	Spexer 2000 3D MkIII Radar
Maximum UAV detection range	9 km	9 km
Maximum small UAV detection range	6 km	6 km
Radar technology	Full coherent pulse Doppler Radar	Full coherent pulse Doppler Radar
Frequency range	X-band	X-band
Azimuth coverage	120°	up to 360° (single antenna 120°)
Elevation coverage	15°	up to 90°
Track while scan	>300 targets	>300 targets in 120°
Power consumption	<550 W	Antenna: 1700 W Processing: 400 W

**Table 4:** Echodyne Counter-Drone Radar specifications.

Specification	Value
Detection range	2.5 km
Frequency	24.05–24.25 GHz
Field of view	120° azimuth 80° elevation
Angular resolution	2° azimuth 6° elevation
Search while track	object tracks are updated at ~10 Hz while continuously scanning entire field of view
Track acquisition rate	<1 s
Max tracks	≤20 simultaneous tracks

**Table 5:** Ranger R8SS-3D specifications.

Specification	Value
Instrumented range	7800 m
Micro-UAV detection range	1200 m
Mini-UAV detection range	2100 m
Small UAV detection range	4000 m
Minimum detection range	10 m
Scan sector	±45° (fixed)–360° with pan/tilt mount
Vertical coverage	≥40°
Number of simultaneously displayed tracks	Up to 512
Electronic scan rate	2 Hz or 4 Hz
Minimum detection velocity	<0.1 m/s
Range accuracy	±3 m
Angular accuracy (azimuth)	<0.8°
Angular accuracy (elevation)	<3°
Operating frequency	X-band
Connectivity	Ethernet

**Table 6:** Doruk radar specifications.

Specification	Value
Frequency band	X-band
Detection probability	80%
Detection range	6 km
Detection velocity	0.2–100 m/s
Elevation beamwidth	20°
Azimuth accuracy	≤1° (RMS)
Azimuth resolution	≤2°
Azimuth coverage	360°
Range accuracy	≤5 m
Range resolution	≤15 m
Velocity accuracy	≤0.2 m/s
Scanning rate	90 °/s
Target tracks	>200, Track While Scan
Clutter suppression	≥45 dB

**Table 7:** DedroneSensor’s RF sensors’ specifications.

Specification	DedroneSensor RF-160	DedroneSensor RF-360
Range	1.6–5 km (depending on RF interference conditions)	2–5 km (depending on RF interference conditions)
Radio frequency	Omnidirectional, passive detection and classification	Omnidirectional, passive detection, classification and direction-finding

**Table 8:** Dedrone DroneTracker audio detector specifications.

Specification	Value
Range	500 m
Coverage azimuth angle (min–max)	10°–90°
Audio spectrum	0–96 kHz
Microphone range	50–80 m

**Table 9:** Axis Q6215-LE PTZ specifications.

Specification	Value
Image sensor	CMOS
Image sensor size	1/1.9 inches
Range	1000 m
Night vision range	400 m
Min illumination/light sensitivity (color)	0.07 lux
Min illumination/light sensitivity (B/W)	0 lux
Max video resolution	1920 × 1080
Max frames per second	50/60
Focal length	6.7–201 mm
Horizontal field of view (min–max)	2.2°–58.6°
Vertical field of view (min–max)	1.2°–34.1°
Pan range	360°
Tilt range	−90° to +90°
Optical zoom	30
Digital zoom	21

**Table 10:** Triton PT-Series HD camera specifications.

Specification	Value
Range	2–4 km (depending on visibility conditions)
Min illumination/light sensitivity (color)	0.01 lux
Max video resolution	1920 × 1080
Focal length	4.3–129 mm
Field of view (min–max)	21° × 28° W 1.5° × 2° N
Lens field of view (min–max)	2.3°–63.7°
Pan range	360°
Pan velocity	0.1 to 60°/s
Tilt range	−90° to +90°
Tilt velocity	0.1 to 30°/s
Optical zoom	120
Digital zoom	22

**Table 11:** ARMS camera specifications.

Optronic
Camera model (IR and CCD) selectable from a wide range
360° PTZ platform
Wide and narrow FoV continuous zoom
Tracking and 3D positioning

**Table 12:** Spynel series specification.

Specification	SPYNEL-C 1000	SPYNEL-S 2000	SPYNEL-X 3500
Horizontal field of view	360°	360°	360°
Vertical field of view	20°	20°	20°
Frame rate	Up to 2 Hz	Up to 2 Hz	Up to 2 Hz
Spectral band	LWIR (8–12 µm)	MWIR (3–5 µm)	MWIR (3–5 µm)
Image resolution	3 Mpixel	7 Mpixel	30 Mpixel
Range	400 m	400 m	400 m

**Table 13:** DroneSentry integrated system specifications.

Specification	Value
Radar detection range	1.5 km
RF detection range	1 km (urban) 5 km (rural)
Acoustic detection range	200 m
Camera detection range	600 m (small UAVs) 2 km (large UAVs)
RadarZero field of view	≥90° azimuth × 80° elevation
RadarZero angle resolution	±1° azimuth ± 3° elevation
RadarZero frequency	24.45–24.65 GHz (multichannel)
RadarZero target detection	≥20 targets simultaneously
DroneOpt pan rotation	360° continuous
DroneOpt pan speed	0.2°/s–120°/s
DroneOpt tilt range	−55°–+90°
DroneOpt tilt speed	0.2°/s–90°/s
DroneOpt position accuracy	±0.07°
DroneOpt zoom	30× optical zoom 12× digital zoom
DroneOpt field of view (min–max)	2.3°–63.7°
DroneOpt resolution	640 × 480
DroneOpt frame rate	30 Hz

**Table 14:** Comparison of UAV sensing technologies.

Method	Operational Conditions	Range	Cost	Measures Provided
Active radar	Partially affected by weather conditions	Long-range (~5 km)	High-cost	Range, azimuth, elevation
Passive radar	Partially affected by weather conditions	Long-range (~5 km)	Low-cost	Range, azimuth, elevation
Radio frequency sensor	Affected by RF interferences and partially by weather conditions	Medium-range (~2 km)	Low-cost	Azimuth, elevation, classification
Acoustic sensor	Affected by weather/noise conditions	Short-range (~500 m)	Low-cost	Azimuth, classification
Camera sensor	Affected by weather conditions, day/night	Medium-range (~1 or 2 km)	Low-cost	Azimuth, elevation, classification
